# Six-month trajectory of physical function in ICU survivors: Experience from an Eastern Cape centre

**DOI:** 10.4102/sajp.v81i1.2228

**Published:** 2025-08-12

**Authors:** Elizabeth van der Merwe, Louise Stroud, Gary Sharp, Noline van Vuuren, Fathima Paruk

**Affiliations:** 1 Department of Critical Care, Faculty of Health Sciences, Nelson Mandela University, Gqeberha, South Africa; 2 Adult Critical Care Unit, Livingstone Hospital, Gqeberha, South Africa; 3 Department of Psychology, Faculty of Health Sciences, Nelson Mandela University, Gqeberha, South Africa; 4 Department of Statistics, Faculty of Science, Nelson Mandela University, Gqeberha, South Africa; 5 Department of Health Sciences, Faculty of Health and Enviromental Sciences, Central University of Technology, Bloemfontein, South Africa; 6 Department of Critical Care, Steve Biko Academic Hospital, Pretoria, South Africa; 7 Department of Critical Care, Faculty of Health Sciences, University of Pretoria, Pretoria, South Africa

**Keywords:** critical care, physical impairment, ICU-acquired weakness, Medical Research Council score, six-minute walk test, health-related quality of life, post ICU pain, post ICU syndrome

## Abstract

**Background:**

Physical impairment affects up to 60% of intensive care unit (ICU) survivors due to factors such as ICU-acquired neuromuscular weakness (ICU-AW), chronic pain, deconditioning and reduced organ and metabolic function. This impairment is linked to lower health-related quality of life (HRQOL).

**Objectives:**

Our study aimed to assess physical impairment and HRQOL among critically ill patients post hospital discharge.

**Method:**

Intensive care unit survivors were assessed six weeks and six months post hospital discharge. Physical performance was evaluated using the six-minute walk test (6MWT) and muscle strength with the Medical Research Council (MRC) score. Patients’ HRQOL was determined using the Rand Short Form-36 questionnaire.

**Results:**

A total of 107 patients (median age 42 years), including 50% with COVID-19, completed the 6-month follow-up. Although significant improvements were observed, 53.5% walked less than 80% of the predicted 6MWT distance at six months, with females disproportionately affected. Poor physical performance was associated with lower physical and mental HRQOL. Pain interfering with activities was reported by 26.2% at six months. Only 2% met full criteria for ICU-AW at six months. By six weeks, only 15% had attended physiotherapy.

**Conclusion:**

Intensive care unit survivors exhibited a high incidence of physical impairment and pain at six months, impacting HRQOL. Very few patients met full ICU-AW criteria.

**Clinical implications:**

Physical impairment after critical illness is multifactorial and is not only attributable to muscle weakness. The recovery process of young, previously non-frail ICU survivors in the public healthcare setting may be improved by introducing rehabilitation pathways.

## Introduction

Surviving critical illness often results in long-term physical disability, a key aspect of the post-intensive care unit (ICU) syndrome. This may be attributed to any combination of ICU-acquired neuromuscular weakness (ICU-AW), chronic pain, deconditioning, reduced organ and metabolic function, and may impact negatively on health-related quality of life (HRQOL) (Herridge et al. 2003; Herridge et al. 2011; Needham et al. 2012). The ICU -acquired neuromuscular weakness is a syndrome of limb and respiratory weakness that frequently develops in the wake of critical illness as a result of the effects of disuse, critical illness, and certain treatments, on motor nerves and skeletal muscle (Kramer 2017).

The incidence of new physical impairments after ICU discharge is unclear, and studies often do not consider preexisting impairments, which may lead to the overestimation of the effects of critical illness on physical function. Up to 60% or more of ICU survivors report new physical disabilities in the year after ICU discharge (Geense et al. 2021).

Identifying and grading physical impairment after ICU admission is important because it directs referral to appropriate rehabilitative care and informs the counselling of patients and families (Fan et al. 2014a). The diagnosis is also crucial for communication with employers and insurers regarding sick leave and disability, as well as for guiding life decisions (Fan et al. 2014a).

The objective of our study was to investigate the trajectory of physical performance of ICU survivors after hospital discharge and its impact on their HRQOL. Our study builds on previous South African research among public sector patients that explored physical impairment and recovery after critical illness (Van Aswegen et al. 2008, 2010, 2011).

## Research methods and design

This single centre prospective observational cohort study screened and enrolled critically ill patients either at ICU discharge or in the ward shortly thereafter. The screening and enrolment was done by a clinical technologist not involved in their care. It constituted a component of a broader study that investigated the physical, mental and cognitive after-effects among ICU survivors. The research was conducted in a multi-disciplinary, closed care, tertiary-level adult critical care unit with 16 beds. Inclusion criteria comprised age ≥ 18 years and the requirement of ≥ 48 h admission to ICU, either for respiratory support (invasive or non-invasive mechanical ventilation or high flow humidified nasal oxygen [HFNO]), or shock requiring vasopressor support, or management for any other organ failure. Patients were excluded if any of the following factors were present: they were prisoners, had a life expectancy gauged to be less than 6 months, resided more than 300 km from the enrolling centre, were unable to be interviewed in English or IsiXhosa, had active or recent psychosis, were admitted for self-harm or with neurological pathology, or had preexisting cognitive impairment (as determined by the Short Informant Questionnaire on Cognitive Decline in the Elderly [IQCODE]) (Quinn et al. 2014). Patients were also excluded from the final data analysis if they did not survive until the 6-month follow-up or did not complete the 6-month study assessment (Van Der Merwe et al. 2024).

Data were collected by the clinical technologist and three specialists in the ICU. Data collected at enrolment included demographic and clinical information. Premorbid clinical frailty was determined using the Clinical Frailty Scale score (CFS) (Rockwood et al. 2005). The patients’ HRQOL was determined by completing the RAND-corporation Short Form-36 HRQOL questionnaire, hereafter referred to as the SF-36 (RAND-Corporation). The SF-36 comprises eight scale scores namely, physical functioning (PF), bodily pain (BP), role limitations because of physical health problems (RP), role limitations because of personal or emotional problems (RE), general mental health (MH), social functioning (SF), energy, fatigue or vitality (VIT) and general health perceptions (GH). These are aggregated to calculate two sub scores, namely, the physical component summary score (PCS) and the mental component summary score (MCS) (RAND-Corporation). To limit barriers like eyesight and reading skills, and to improve completeness, all questionnaires were clinician administered. If patients preferred to be interviewed in isiXhosa, or when the clinician observed discrepancies using the English SF-36 version, an isiXhosa-speaking ICU physician conducted the interview using a certified isiXhosa translation. Physical impairment was assessed with the six-minute walk test (6MWT) distance. A standardised protocol, described in the American Thoracic Society Statement, was used to administer this test over a 30 m distance (2002). Poor physical performance was defined as less than 80% of the predicted distance (Herridge et al. 2011). The predicted 6MWT distance was calculated using an equation which adjusts for age, height, gender and weight (Enright & Sherrill 1998). The minimum clinical important distance for the 6MWT is considered to be no less than 30 m in ICU survivors (Chan et al. 2015).

Muscle weakness was assessed with the Medical Research Council (MRC) score. The MRC uses manual testing of the strength of various muscle groups in the upper and lower extremities to grade muscle strength (Fan et al. 2014a). It scores each muscle group’s strength from zero to five, with higher scores indicating greater muscle strength (Fan et al. 2014a; Kramer 2017). The total MRC score is calculated by adding the power in six main muscle groups of the upper and lower limbs, and in the absence of other causes for weakness, a value of less than 48 out of a maximum 60 is diagnostic of ICU-AW (Fan et al. 2014a; Kress & Hall 2014).

Continuous data were tested for normality using the Kolmogorov–Smirnov test. Normally distributed data are reported as mean and standard deviation (SD) and skewed data as median and interquartile range (IQR). Binary data are presented as numbers and percentages. The *t*-tests for dependent and independent samples, Mann–Whitney *U* test, Wilcoxon signed-rank test with continuity correction were used to compare continuous data (Van Der Merwe et al. 2024). For comparison of binary variables, the Chi-square and Fischer’s exact tests were used as appropriate. Bivariate analysis compared variables between patients with good and poor physical performance at 6 months. In all cases, the level of significance is at 5%. All analyses were performed using *R* (version 4.2.2), The *R* Foundation for Statistical Computing, Vienna, Austria.

### Ethical considerations

Informed consent was sought from patients or alternatively their surrogate decision-makers either at ICU discharge or shortly thereafter in the ward, depending on when it was most feasible. Where a surrogate decision-maker provided the initial assent, consent was obtained from the patient when the patient regained the capacity to consent. Permission for our study was obtained from the Nelson Mandela University’s Research Ethics Committee (Human) (reference number: H20-HEA-PSY-001) on 01 May 2020.

## Results

**FIGURE 1:**
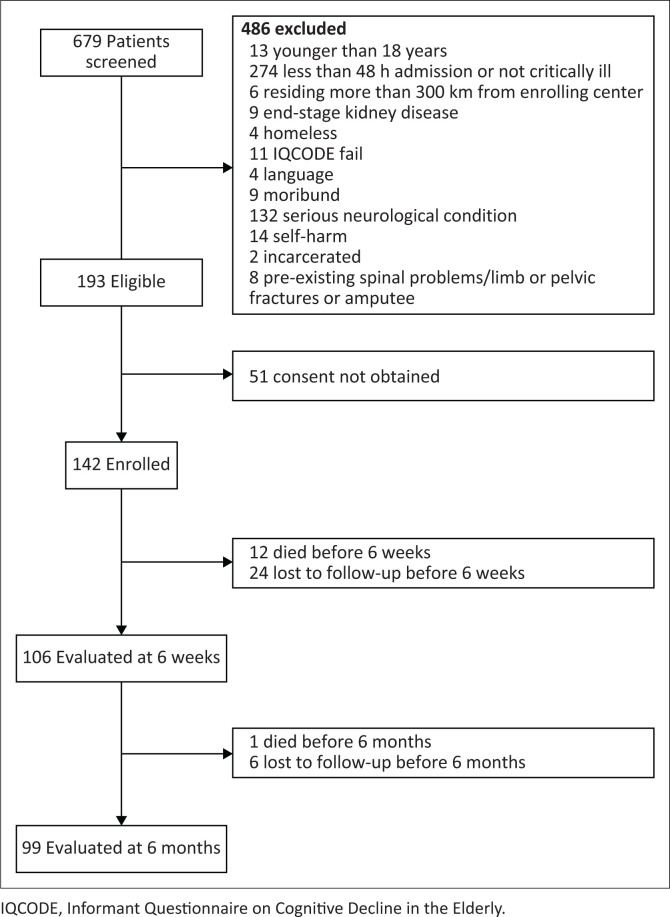
Process of screening and inclusion.

Enrolment and data collection commenced on the 01 August 2020, just after the peak of the first local coronavirus disease 2019 (COVID-19) pandemic wave, and was completed on the 31 May 2022. There was a 3-week hiatus in the enrolment during December 2020, owing to the investigators contracting COVID-19. Our study period also included the second and third waves of the pandemic. The process of screening and inclusion set in Figure 1 (Van Der Merwe et al. 2024).

A comparison of the main characteristics (sex, age, diagnostic category, work status, severity of disease, frailty and co-morbidities and length of ICU stay) between our study group and those lost to follow-up did not show any significant differences (Appendix 1, Table 1-A1).

At the 6-week study visit, of the 50 ICU survivors with COVID-19-related admissions, 11 (22%) were referred to the respiratory clinic for further investigations for ongoing respiratory symptoms. The clinical characteristics of the 99 patients who were able to complete the full physical assessment at 6 months are set out in Table 1.

**TABLE 1:** Clinical characteristics of cohort.

Clinical characteristics	Cohort *N* = 99				Good physical performance *n* = 46				Poor physical performance *n* = 53				*p*-value
	*n*	%	Median	IQR	*n*	%	Median	IQR	*n*	%	Median	IQR	
**Sex**	-	-	-	-	-	-	-	-	-	-	-	-	0.065
Female sex	55	55.6	-	-	21	45.7	-	-	34	64.2	-	-	-
Male sex	44	44.4	-	-	25	54.3	-	-	19	35.8	-	-	-
**Age (years)**	-	-	40	33–51	-	-	45	37–57	-	-	36	31–48	0.019*
**Clinical Frailty Scale**	-	-	2	2–2	-	-	2	2–2	-	-	2	2–2	0.864
**Charlson Comorbidity Index**	-	-	0	0–0	-	-	0	0–0	-	-	0	0–0	0.677
**SAPS 3 score**	-	-	48	44–55	-	-	48	42–53	-	-	48	44–55	0.434
**Highest SOFA score,**	-	-	4	4–9	-	-	4	4–7	-	-	5	4–9	0.626
**Hospital days**	-	-	23	14–36	-	-	24	13–39	-	-	23	14–34	0.814
Hospital stay longer than 7 days	93	93.9	-	-	41	89.1	-	-	52	98.0	-	-	0.094
**ICU days**	-	-	7	5–14	-	-	7	6–13	-	-	8	5–17	0.836
ICU stay longer than 7 days	48	48.5	-	-	20	43.5	-	-	28	52.8	-	-	0.353
ARDS	72	72.7	-	-	36	78.2	-	-	36	67.9	-	-	0.250
PF ratio	-	-	115	82–222	-	-	93	67–213	-	-	130	94–228	0.080
Invasive ventilation	44	44.4	-	-	16	34.8	-	-	28	52.8	-	-	0.070
Ventilated longer than 7 days	18	18.2	-	-	6	13.0	-	-	12	22.6	-	-	0.217
COVID-19 related admission	50	50.5	-	-	30	65.2	-	-	20	37.7	-	-	0.006*
**Diagnostic category**													
Surgical	21	21.2	-	-	7	15.2	-	-	14	26.4	-	-	0.174
Medical†	61	61.2	-	-	34	73.9	-	-	27	50.9	-	-	0.019*
Trauma	17	17.1	-	-	5	10.9	-	-	12	22.6	-	-	0.121
**Complications and treatments**													
Sepsis	83	83.3	-	-	40	90.0	-	-	43	81.1	-	-	0.432
Septic shock	24	24.2	-	-	8	17.4	-	-	14	26.4	-	-	0.281
Acute kidney injury	52	52.5	-	-	21	45.7	-	-	31	58.5	-	-	0.202
Vasopressor treatment	28	28.3	-	-	12	26.1	-	-	16	30.2	-	-	0.651
Non-depolarising muscle relaxants	5	5.1	-	-	2	4.3	-	-	3	5.7	-	-	-
Corticosteroid treatment	70	70.7	-	-	38	86.6	-	-	32	60.4	-	-	0.138
Corticosteroid treatment > 7 days	30	31.3	-	-	13	28.2	-	-	18	34.0	-	-	0.541

HFNO, high flow humidified nasal oxygen therapy; fiO_2_, fraction of inspired oxygen; NIV, non-invasive ventilation; SAPS, simplified acute physiology score; SOFA, sequential organ failure score, PF, arterial oxygen/fraction of inspired oxygen; ARDS, acute respiratory distress syndrome; ICU, intensive care unit; COVID-19, coronavirus disease 2019.

* , statistical significant at *p* < 0.05.

† , Medical admissions included the COVID-19 cases.

Physical impairment was measured with the 6MWT. The median (IQR) meters (m) that could be walked by the cohort at 6 weeks and 6 months were 405 (315–478) and 470 (390–518), (*p* < 0.001), respectively. The median percentage of predicted distance walked at 6 weeks and 6 months was 70.9% and 79.9%, respectively. At 6 weeks, 79 (79.8%) patients walked less than 80% of the predicted distance, while at 6 months, 53 (53.5%) patients walked less than 80% of predicted. The poor outcome group (i.e. those who walked < 80% of predicted) walked a median of 69.2% of the predicted distance at 6 months, while the good outcome group (≥ 80% of predicted) walked a median of 89.9% of predicted. The distances walked at 6 weeks and 6 months by males and females in the two outcome groups are reported in Table 2. Among those whose physical performance was classified as poor, all males walked less than 590 m, and females walked less than 540 m with only three of the females exceeding 500 m.

**TABLE 2:** Comparisons of six-minute walk test distances.

Variables	Poor outcome group				Good outcome group				*p*-value
	*n*	%	Median	IQR	*n*	%	Median	IQR	
**Female participants**	55	-	-	-	-	-	-	-	-
6-week visit	46	83.6	347	188–408	9	16.4	430	418–465	0.003
6-month visit	34	61.8	365	305–424	21	38.2	480	450–500	< 0.001
**Male participants**	44	-	-	-	-	-	-	-	-
6-week visit	33	75.0	420	360–493	11	25.0	540	469–564	0.002
6-month visit	19	43.2	480	429–505	25	56.8	540	510–587	0.001

Note: The unit measurement for the median and interquartile range numbers is meters.

IQR, interquartile range.

**FIGURE 2:**
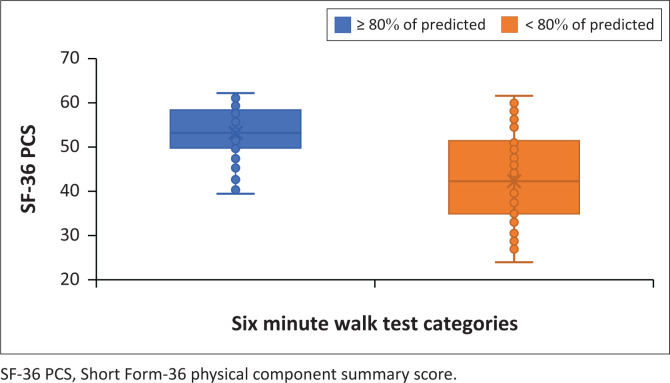
Short Form-36 physical component summary score for six-minute walk test categories.

**FIGURE 3:**
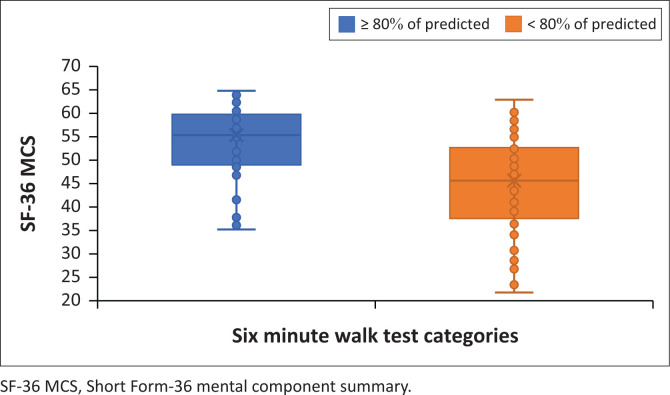
Short Form-36 mental component summary score for six-minute walk test categories.

Those who walked less than 80% of the predicted 6MWT distance at 6 months had a significantly lower median (IQR) SF-36 PCS than those who walked 80% or more of the predicted (42.1 [35.6–51.3] vs 53.3 [49.8–58.4], *p* < 0.001) (Figure 2). Those who walked less than 80% of predicted also had a significantly lower median (IQR) SF-36 MCS than those who walked 80% or more of the predicted (45.6 [37.6–52.7] vs 55.2 [49.1–59.75], *p* < 0.001) (Figure 3).

At 6 weeks, only three patients (3%) had criteria for ICU-AW, namely an MRC score of lower than 48. However, 66 (66.7%) patients had lower than normal muscle power at this same time point. Similarly, while only two patients (2%) had an MRC score lower than 48 at the 6-month visit, 34 (34.3%) had lower than normal muscle power at 6 months. Those who were found to have any muscle weakness at 6 months had a significantly lower median (IQR) SF-36 PCS as compared to those who had normal muscle power (45.8 [37.0–52.2] vs 51.2 [42.2–56.8], *p* = 0.039). Of the 53 patients who walked a poor 6MWT distance at 6 months, 30 had no detectable muscle weakness as indicated by an MRC score of 60.

A significant proportion of patients still experienced pain at both visits, and this frequently interfered with their daily activities and work (Table 3). Only five of the patients with moderate to very severe pain at 6 months had physical trauma as the reason for ICU admission.

**TABLE 3:** Pain at 6 weeks and 6 months after hospital discharge.

Study visits	Moderate, severe or very severe pain		Pain interfering moderately, quite a bit or extremely with activities	
	*n*	%	*n*	%
6-week visit	34	31.8	36	33.6
6-month visit	23	21.5	28	26.2

Sixteen (15.0%) patients had attended outpatient physiotherapy after hospital discharge, and none had received occupational therapy.

Table 1 compares the demographic and clinical characteristics of the patients with a good and poor physical performance. Multiple logistic regression analysis utilised variables that had a *p*-value of 0.1 or lower on bivariate analysis, as well as corticosteroid treatment (a previously described association with ICU-AW). Age, female sex, surgical or trauma admission diagnosis (as opposed to medical diagnosis), a non-COVID-19 admission diagnosis, PF ratio, invasive ventilation, corticosteroid treatment and a hospital stay > 7 days were included in the final logistic analysis model. Independent associations with physical performance were determined with backwards stepwise analysis (Table 4). Predictors of poor performance in this model were female sex, a non-COVID-19-related admission diagnosis and, paradoxically, a younger age.

**TABLE 4:** Predictors included in multiple logistic regression analysis.

Patient variables	aOR	95% CI	*p*-value
Age, per year	0.95	0.91 – 0.99	0.013*
Female sex	3.7	1.6 – 13.5	0.011*
Non-COVID-19 admission diagnosis	10.3	2.9 – 43.5	0.001*
PF ratio per 1 mmHg	0.996	0.991 – 1.001	0.090
Hospital stay > 7 days	10.3	1.2 – 245.4	0.066

COVID-19, coronavirus disease 2019; PF, arterial oxygen/fraction of inspired oxygen; aOR, adjusted odds ratio; CI, confidence interval.

* , statistical significant at *p* < 0.05.

In view of unexpected finding related to age, further analyses of COVID-19 versus non-COVID-19 patients were performed. First of all, at 6 months, patients with COVID-19 performed significantly better as compared to non-COVID-19 patients, as witnessed by a median (IQR) percentage of predicted distance of 83 (77.6–95.0) versus 74 (61.7–81.5), respectively, *p* < 0.001. In keeping with this is the fact that among those in the cohort with a good physical outcome, there were more COVID-19 than non-COVID-19 patients (30 vs 16). The COVID-19-related admissions with a good outcome had a significantly higher median (IQR) age than non-COVID-19 patients in the same physical performance group (47 [38–55] vs 39 [33–58], *p* = 0.045).

## Discussion

We observed that more than half of the relatively young ICU survivors, who had few comorbidities and low frailty, walked shorter than the expected distance 6 months after hospital discharge. This impacted negatively on both their physical and mental HRQOL.

Chronic pain after ICU admission is multifactorial and includes increased levels of preexisting chronic pain, chronic systemic inflammation, medical procedures or prolonged immobilisation (Baumbach et al. 2016). Only five of the patients experiencing moderate to very severe pain at 6 months had an admission diagnosis of physical trauma. This underscores the multifactorial nature of post ICU pain. While there was an improvement in pain between the 6-week and the 6-month visit, 26% of patients still experienced ongoing pain that interfered with their activities at 6 months. This finding is in keeping with the findings of a recent systematic review which reports that the incidence of persistent pain 6 months post discharge ranges between 22% and 44% (Mäkinen et al. 2020). In the Eastern Cape, where many are dependent on their physical health to perform remunerative work and where public transport is underdeveloped, pain interfering with activities may mean the difference between unemployment and employment for ICU survivors.

Although there is no consensus regarding the optimal assessment and screening tools for general physical impairment post ICU, the 6MWT exhibits the highest rating among experts as a performance-based test for survivors of respiratory failure and critical illness (Major et al. 2016; Needham et al. 2017). During the 6MWT, the cohort walked a mean of 79.9% of the predicted distance at the 6-month study visit, which is better than the 58% – 72% reported in prepandemic studies from higher resourced countries (Dinglas et al. 2016; Fan et al. 2014b; Herridge et al. 2003; Li et al. 2006; Masclans et al. 2011; Needham et al. 2013; Pfoh et al. 2016; Wright et al. 2009). The relatively high percentage of predicted distance walked by our total cohort is likely because of the inclusion of the COVID-19 patients, who performed better as compared with non-COVID-19 patients (83% of predicted distance vs 74%). This is in keeping with the findings of other studies among COVID-19 ICU survivors. The subgroup of *non*-COVID-19 patients in our study walked 74% of the predicted distance at 6 months, which is more aligned to the range previously reported in non-COVID-19 cohorts.

Despite the improvements from the 6-week to the 6-month visits, it is sobering to note that around 80% of the patients in our study walked less than 80% of the predicted distance at 6 weeks, and at 6 months, this was still the case for over 50% of the cohort. Those classified as having a poor physical performance only walked a median of 69% of predicted distance at 6 months. Of particular concern is the fact that 62% of females had a poor performance at 6 months, and that the subgroup had not made significant progress (less than the minimum clinically significant distance). The only previous South African study for comparison enrolled young males with penetrating trunk trauma and report median distances of 628 m – 680 m at 6 months, which was similar to that of a healthy control group (Van Aswegen et al. 2010).

The lack of South African predicted values for the 6MWT is a limitation and may have led to misclassification of some patients in our study. However, it is important to adjust for certain factors like sex and age when reporting on 6MWT distances, and other South African authors have also used international prediction models (Roos et al. 2022). Six-minute walk test distances walked by healthy adults aged 20–50 years in Australia and the USA are around 590 ± 60 m for females and 640 ± 40 m for males (Chetta et al. 2006). These distances correlate with the distances reported in healthy volunteers from other parts of the world (2002) and also distances walked by older volunteers (Troosters, Gosselink & Decramer 1999). Locally, a healthy male control group (mean age around 30 years) all walked further than 600 meters (Van Aswegen et al. 2010). If one considers the distances walked by healthy volunteers in SA and further afield, then the finding that all the males in our study labelled as poor walked less than 590 meters, and females less than 540 meters, likely implies that they were accurately classified as physically impaired. Furthermore, those labelled as poor had a markedly lower physical and mental HRQOL as compared to the good physical outcome group.

Very few patients met the full criteria for ICU-AW at the study visits. This was in keeping with the findings of other studies that, despite marked reductions in the 6MWT, only 4% – 15% of patients had MRC score < 48/60 at 6 months (Fan et al. 2014b; Needham et al. 2014). Although some ICU survivors suffer from the consequences of ICU-AW for many years (Batt et al. 2013; Herridge et al. 2011; McHugh et al. 1994), previously healthy, non-frail ICU survivors, like in our study, may recover quicker (Dowdy et al. 2005; Herridge et al. 2003, 2011, 2016). However, the negative effect on HRQOL was notable in those in our study cohort who had any degree of neuromuscular weakness on follow-up, even when full ICU-AW criteria were not met.

The 6MWT distance serves as a general screening test for physical impairment but is nonspecific and nondiagnostic; therefore, further investigation is warranted to define the contributions of cardiac, respiratory, neuromuscular and other pathology (2002). Of the patients who walked less than 80% of the predicted distance in the 6MWT, 30 patients (56.6%) had no detectable muscle weakness on clinical examination. This finding underscores that physical impairment in ICU survivors is multifactorial and includes muscle endurance (rather than just strength), cardiorespiratory and metabolic function, pain and psychological status. In view of these findings, we concur with others that limb muscle strength alone, and particularly the 48/60 MRC score diagnostic cut-off for ICU-AW, should not be used in isolation to screen for, or determine, global physical impairment after hospital discharge (Needham et al. 2014; Ohtake et al. 2018).

Experts agree that there should exist a continuum of personalised rehabilitation that extend beyond hospital discharge (Herridge & Azoulay 2023; Major et al. 2016; Mikkelsen et al. 2020). It is therefore alarming that only 15% of our cohort were receiving outpatient physiotherapy at the 6-week visit and that none had occupational therapy. The reasons for this were not explored in this research. Allied health services in the local setting were curtailed during the initial pandemic waves, and this may have been a contributory factor. It is the authors’ impression that there is scope for research on the barriers to personalised multidisciplinary rehabilitation after critical illness in SA.

Significant associations with poor physical performance included female sex, a non-COVID-19 admission diagnosis and, paradoxically, older age. These findings will be discussed in the following paragraphs.

A recent systematic review of eight studies on physical impairment post ICU also found female sex to be a risk factor for poor physical performance, and that ICU-related variables, namely, severity of illness or mechanical ventilation, had no consistent association (Parry et al. 2021). Overall, the lack of consistent independent associations related to disease severity across various studies, points to the complexity of the pathogenesis of physical impairment in ICU survivors and that pathology in various organ systems may account for it. Unlike the findings of the systematic review (Parry et al. 2021), pre-existing comorbidity was not associated with a higher incidence of physical impairment in our study. The lack of association may be explained by the cohort’s overall low baseline frailty and comorbidity index.

In the same prepandemic review of physical impairment (Parry et al. 2021), ICU survivors who experienced the complication of ARDS were found to walk significantly shorter distances than those without ARDS. Prepandemic studies used the Berlin definition for ARDS (Ferguson et al. 2012), which includes the requirement for at least five centimetres of water of positive end expiratory pressure (PEEP) administered with a ventilator. In our study, the recently proposed post COVID-19 criteria for ARDS, which includes high flow nasal oxygen therapy with flow > 30 L/min, was used (Matthay et al. 2024). Most of the cases of ARDS in our study were because of COVID-19 (69%), and most of these survivors only required non-invasive respiratory support, whereas all the non-COVID-19 Acute respiratory distress syndrome (ARDS) cases had to be intubated and ventilated. Non-invasive respiratory support has been found to be a reasonable first step in the management of COVID-19-related ARDS, and patients who recovered after only requiring non-invasive ventilation have been shown to have a significantly lower mortality than those requiring intubation (Calligaro et al. 2020; Nevola et al. 2022). In one study, physical and HRQOL outcomes were similar for COVID-19 patients who were intubated and ventilated compared with those who only received non-invasive ventilatory strategies (Schandl et al. 2021). As such, the COVID-19 pneumonia caused by the initial viral variants of the pandemic culminated in a unique form of ARDS, with different outcomes and disease trajectories than ARDS caused by other conditions. This may explain the finding in our study that ARDS was not independently associated with physical impairment.

Although some COVID-19 ICU survivors also report to suffer from significant post-intensive care unit syndrome (PICS) symptoms, there are only a few studies that report upon objective measurements, for example, the 6MWT, for comparison. Those studies that did use the 6MWT report distances at 6 months ranging between 70% and 88% of predicted, and this aligns with our study’s finding that physical performance was better among COVID-19 ICU survivors as compared to the non-COVID-19 ICU survivors (Carenzo et al. 2021; Huang et al. 2021; Latronico et al. 2022; Schandl et al. 2021). The incidence of ICUAW, as measured by the 48/60 MRC score cut-off, also reports to be very low in COVID-19 ICU survivors (less than 2%) (Latronico et al. 2022). Studies assessing physical disability in COVID-19 ICU survivors in a variety of ways report physical disability rates that tend to be lower or similar than that reported in non-COVID-19 ICU survivors, and COVID-19 patients may recover and return to work faster (Piva et al. 2023; Rasulo, Piva & Latronico 2021).

In a meta-analysis that included data from higher income countries, older age is generally associated with greater physical impairment (Parry et al. 2021), and therefore the finding of our study that older age is associated with better age-corrected physical outcomes was unexpected. There are certain considerations in this regard. Firstly, our study cohort was generally non-frail and young (less than a third of patients were 50 or older), and within this age category of ICU patients, older age alone may not be an independent marker for poor physical outcomes (Herridge et al. 2016). Secondly, on secondary analysis of the good outcome group, it was found that the COVID-19-related admissions dominated the good outcome group and that they were also significantly older as compared to the non-COVID-19 patients, possibly a result of independent variable interactions in the analysis. This phenomenon may reflect the strict process of triaging during the COVID-19 pandemic waves when older patients were more likely to be admitted if they had fewer pre-existing health problems and less organ failure at presentation.

Limitations in our study include the lack of SA population norms for the 6MWT and concerns regarding the subjective distinction between the MRC scale scores 4 and 5, as well as its ability to detect subtle changes due to its ordinal nature. (Connolly et al. 2013; Hermans et al. 2014; Vanhorebeek, Latronico & Van Den Berghe 2020). The COVID-19 pandemic may as such have introduced various confounders in our study. This included the unique form of COVID-19-related ARDS, the high mortality among those who were intubated and triaging of referrals at the height of the pandemic waves. It also meant that we did not reach our aim of including more patients with trauma, surgical emergencies and HIV disease. However, this is the largest study in the field of PICS in SA to date, and we did show that it is possible to follow up ICU patients in South Africa after hospital discharge with a favourable retention rate. This may be attributed to the provision of a transport stipend, phone calls reminding them of their visits and a flexible study visit booking system.

## Conclusion

There was a high incidence of physical impairment among ICU survivors at 6 months. Female patients were more affected by physical impairment after ICU admission, and very few patients received physio- and occupational therapy directly after hospital discharge. More than a quarter of patients still experienced pain at 6 months, and this had a significant impact on their activities. Physical impairment at 6 months after hospital discharge was associated with a poorer physical and mental HRQOL. Very few patients met the full criteria from ICU-AW. Similar to other authors, we conclude that limb muscle power should not be used in isolation to screen for physical impairment in ICU survivors. There is considerable scope for research into rehabilitation needs and services in critical care survivors in South Africa.

## Appendix 1

**TABLE 1-A1:** Comparison between those lost to follow-up and the study cohort.

Variables	Lost to follow-up (*n* = 30)				Study cohort (*n* = 107)				*p*-value
	*n*	%	Median	IQR	*n*	%	Median	IQR	
Female	16	53.0	-	-	58	54.2	-	-	0.902
Age	-	-	37	30–46	-	-	42	33–51	0.116
Remunerative work	16	53.0	-	-	68	63.6	-	-	0.310
Clinical Frailty Scale score	8	26.7	2	2–3	22	20.6	2	2–2	0.873
SAPS 3 score	8	26.7	47	41–54	21	19.6	48	43–55	0.358
Days in ICU	14	46.7	6	5–10	64	59.8	7	5–14	0.084
**Diagnostic category on admission**	-	-	-	-	-	-	-	-	0.436
Trauma versus	8	26.7	-	-	22	20.6	-	-	-
Surgical versus	8	26.7	-	-	21	19.6	-	-	-
Medical diagnosis	14	46.7	-	-	64	59.8	-	-	-

IQR, interquartile range; SAPS, simplified acute physiology score; ICU, intensive care unit.
